# The role of ASPECTS score, atrial fibrillation, and revascularization therapy in predicting hemorrhagic transformation of ischemic stroke: results from a prospective cohort study

**DOI:** 10.3389/fneur.2025.1624711

**Published:** 2025-07-23

**Authors:** Elena Costru-Tasnic, Mihail Gavriliuc, Olesea Odainic, Mihai Tasnic, Elena Manole

**Affiliations:** ^1^Diomid Gherman Institute of Neurology and Neurosurgery, Chisinau, Moldova; ^2^Nicolae Testemitanu State University of Medicine and Pharmacy, Chisinau, Moldova; ^3^MedPark International Hospital, Chisinau, Moldova

**Keywords:** hemorrhagic transformation, ischemic stroke prognosis, aspects, atrial fibrillation, revascularization therapy

## Abstract

**Introduction:**

Hemorrhagic transformation (HT) can seriously complicate and worsen the clinical outcome of acute ischemic stroke (AIS) patients. Atrial fibrillation (AF), larger stroke volumes, and reperfusion therapy are associated with increased risk for HT in AIS. The aim of our research was to evaluate the combined prognostic value of the ASPECTS score, AF, and revascularization therapy in predicting HT in AIS patients.

**Methods:**

A prospective observational study was conducted. Demographic data, presence of AF, application of revascularization therapy, and baseline ASPECTS were recorded. The primary outcome was the occurrence of HT during hospitalization. Univariate and multivariate logistic regression analyses were applied to identify potential predictors. ROC analysis was performed to assess the prognostic value of the analyzed parameters for HT prediction.

**Results:**

Data from 150 successive AIS patients was registered for the final analysis. The active group (with HT during hospitalization) included 55 patients. No significant differences in age, gender distribution, or time to admission between the HT and control groups, were recorded. Lower ASPECTS, presence of AF, and revascularization therapy were independently associated with an increased risk of HT (*p* = 0.001, *p* = 0.004, and *p* = 0.007, respectively). ROC analysis for combined use of ASPECTS, AF, and revascularization therapies showed good predictive performance, AUC 76.51% (95% CI: 68.16–84.86%).

**Conclusion:**

Our findings suggest that ASPECTS score, AF, and revascularization therapy are important predictors of hemorrhagic transformation in AIS. These results support the use of a combined clinical-imaging approach for risk stratification of HT in acute ischemic stroke patients.

## Introduction

Cerebral ischemic stroke represents the second mortality cause and the third reason for disability worldwide (1), representing a major medical and social burden. Given the uncertainties linked to both treatment efficiency, but also post-stroke complications’ possible mechanisms and reliable predictors, extensive research in the field is done to identify markers associated with good/poor post-stroke outcome. Hemorrhagic transformation (HT) of ischemic stroke is among the most serious complications of thrombolytic and endovascular therapies (2). The incidence of HT varies considerably among studies: up to 70% in radiologic and morphologic analyses, approximately 27% in clinical studies, and a relatively stable proportion of symptomatic HT at 5–6% (3–5). Atrial fibrillation (AF) and larger infarct volumes are consistently reported as risk factors and predictors for HT (6). The admission Alberta Stroke Program Early CT Score (ASPECTS) quantifies the early ischemic changes in AIS patients; lower scores have been associated with HT risk (7). Furthermore, the presence of atrial fibrillation (AF) and the revascularization therapy, including intravenous thrombolysis and mechanical thrombectomy, are linked to an increased risk of HT (8, 9). However, the combined predictive value of these parameters remains insufficiently researched in prospective clinical studies.

The aim of our study was to evaluate the combined prognostic value of the Alberta Stroke Program Early CT Score, atrial fibrillation, and revascularization therapy in predicting hemorrhagic transformation in a prospective cohort of acute ischemic stroke patients.

## Methods

We have realized a prospective cohort study to assess the combined prognostic value of the baseline ASPECTS, AF, and revascularization therapy in predicting HT among acute ischemic stroke patients. Secondary outcomes included the evaluation of mortality rates and functional neurological recovery, assessed by the modified Rankin Scale (mRS) at discharge and at 3-month follow-up.

The study included consecutive AIS patients admitted to a tertiary neurological center, within 24 h of symptom onset, between March 2018 and July 2022 (in two phases: 2018–2019 and 2021–2022). The study protocol was approved by the Ethics Committee of the “Nicolae Testemitanu” State University of Medicine and Pharmacy, Chisinau, Republic of Moldova (Approval No. 66, 26 April 2017).

Inclusion criteria: patients aged ≥18 years, presenting with clinical and imaging diagnosis of AIS, admitted within 24 h of symptom onset, and who provided written informed consent. Exclusion criteria: patients <18 years, presence of intracranial hemorrhage on initial brain imaging, and lack of written informed consent.

The number of patients was predefined based on the incidence data and the study protocol. A formal statistical power analysis was not feasible due to the lack of reliable estimates of the expected effect size at the time of study design, which represents a methodological limitation.

An initial number of 176 patients were screened, with 150 being included in the final analysis. From the 26 excluded patients, 5 presented with stroke-mimics, 23 had incomplete diagnosis workout and 3 could not be contacted for 3 months’ follow-up.

Stroke severity was assessed clinically using the NIHSS score, and all patients underwent non-contrast cerebral CT to determine ASPECTS. Data regarding the presence of previously diagnosed AF, the type of oral anticoagulants (OAC) used before admission, hemostasis parameters, stroke subtype according to TOAST classification, and application of intravenous thrombolysis and/or mechanical thrombectomy as revascularization therapies, were collected.

A follow-up CT was performed during hospitalization, between 24 and 72 h after admission or earlier in case of neurological status deterioration, to detect the occurrence of hemorrhagic transformation. HT was established and classified according to ECASS II criteria. Brain imaging was analyzed by a stroke neurologist and confirmed by a neuroradiologist, both blinded to clinical data. Mortality and mRS scores were recorded at discharge and at 3-month follow-up.

Patients were grouped in the active (patients with HT after stroke) and control cohorts (patients without HT during hospitalization). The number of patients in the active group was targeted at 55 cases, by the study protocol.

Beside the variables of interest for HT prediction (AF, ASPECTS and revascularization therapy), other common cardiovascular risk factors like high blood pressure (HBP), diabetes mellitus (DM) were registered and analyzed for potential interactions.

Statistical analysis was performed using R software (version 4.1.1). Group comparisons were conducted using Pearson’s Chi-squared test, Fisher’s exact test, and the Two-Sample t-test. Correlations were evaluated by Pearson’s correlation coefficient. Univariate and multivariate regression models were used to explore associations between analyzed parameters and the risk for occurrence of HT. The studied variables included: baseline NIHSS score, SPAN-100 score, THRIVE score, ASPECTS, high blood pressure, atrial fibrillation, diabetes mellitus, previous antiplatelet use and anticoagulant use, statin use, revascularization therapy, admission glucose, total cholesterol, LDL, platelet count, INR, leukocyte count, and plasma levels of MMP-2 and MMP-9. Variables showing a statistically significant association with HT in the univariate analysis (*p* < 0.05) were subsequently included in a multivariate logistic regression model. These included atrial fibrillation, ASPECTS score at admission, and use of revascularization therapy. Receiver Operating Characteristic (ROC) curve analysis was applied to determine the predictive value of these parameters. A *p*-value < 0.05 was considered statistically significant.

## Results

A total of 176 patients were screened, with 150 acute ischemic stroke patients being included in the final analysis. Patients’ inclusion continued until the target number of hemorrhagic transformation cases—55 patients, as predefined in the study protocol—was reached. The control group included 95 patients without hemorrhagic transformation during hospitalization.

The distribution by HT subtypes in the HT group, according to ECASS II classification, showed a net predominance of the type I of hemorrhagic transformation (hemorrhagic infarction (HI) type 1) (23 patients), followed by a nearly equal number of HI type 2 cases (11 patients) and parenchymal hematoma (PH) type 1 cases (12 patients). The smallest number of cases was for PH type 2 (9 patients). Symptomatic HT, defined as a neurological worsening with a ≥ 4-point increase in the NIHSS score, was recorded in 9 patients (6%).

The general demographic parameters did not different significantly among the studied groups. Therefore, the mean age was 70 years (SD = 10; range: 43–96 years) in the HT group and 71 years (SD = 10; range: 41–94 years) in the control group (*p* = 0.51). Gender distribution was also comparable, with mild female predominance in the HT group (34 of 55 patients (61.8%)), compared to 47 of 95 patients (49.5%) in the control group (*p* = 0.14). Time from clinical symptoms onset to hospital admission was statistically not significantly longer in the control group: 344 min (SD = 278; range: 60–1,200 min) in the HT group and 394 min (SD = 340; range: 60–1,440 min) in the control group.

According to the TOAST classification (*Trial of ORG 10172 in Acute Stroke Treatment*), most patients were classified as cardioembolic stroke (42%), followed by large artery atherosclerosis (36%), stroke of other determined cause (12%), stroke of undetermined cause (5.3%) and lacunar stroke (4.7%). There were significant differences for the classification between the HT and control groups as follows (HT group vs. control group): large artery atherosclerosis – 25% (14 patients) vs. 42% (40 patients); cardioembolic stroke – 53% (29 patients) vs. 36% (34 patients); small vessel disease – 0% (0 patients) vs. 7.4% (7 patients); stroke of other determined etiology – 13% (7 patients) vs. 12% (11 patients); and stroke of undetermined etiology – 9.1% (5 patients) vs. 3.2% (3 patients). The simple logistic regression analysis established that HT was significantly higher in cardioembolic strokes compared to large-artery atherosclerosis ones (*p* = 0.026), and in strokes of undetermined origin compared to large-artery atherosclerosis strokes (*p* = 0.049).

The mean baseline NIHSS score was significantly higher in the HT group (15 points) compared to the control group (11 points; *p* < 0.001). Atrial fibrillation was registered significantly more frequently in the active cohort than in controls (73% versus 47%, *p* = 0.003). All 85 patients were with known atrial fibrillation (40 in the hemorrhagic transformation group and 45 in the control group). Only 23.53% (20 out of 85) were receiving oral anticoagulants (OAC) before admission, the proportion being similar in the HT-group (25%) and the control group (22.22%). All patients were treated with vitamin K antagonists (warfarin), but a therapeutic INR (>2) at admission was registered in just one patient per group. None of the patients were treated with DOACs or low-molecular-weight heparins prior to admission.

The baseline hemostasis parameters showed that mean INR was significantly lower in the HT group (1.27 ± 0.22) compared to control goup (1.38 ± 0.25), *p* = 0.003. However, platelet count (HT group: 228 × 10^9^/L vs. 238 × 10^9^/L, *p* = 0.45) and plasma fibrinogen levels (HT: 3.70 g/L vs. 3.78 g/L, *p* = 0.21) presented no significant differences between the compared groups. Regression analyses in our study found an inverse association between admission INR and the risk of HT (univariate OR = 0.1, 95% CI: 0.02–0.5, *p* = 0.01; multivariate OR = 0.9, 95% CI: 0.01–0.75, *p* = 0.033).

**Figure 1:**
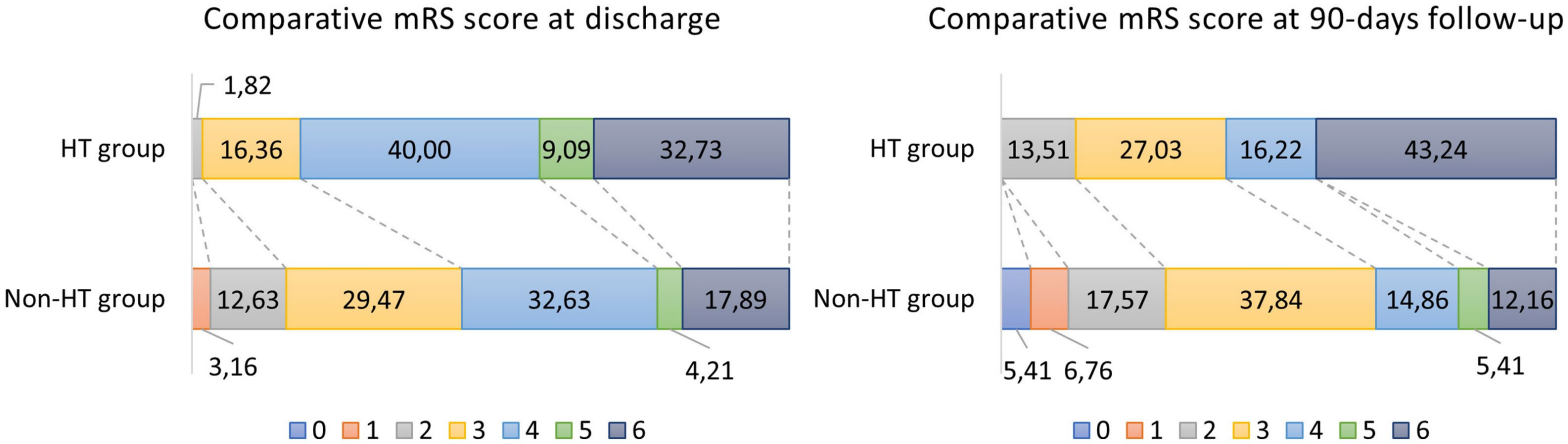
Distribution of modified Rankin Scale (mRS) scores in patients with and without hemorrhagic transformation (HT), assessed at discharge and at 90-days follow-up. Poor functional outcome (mRS 3–6) was more frequent in the HT group at both time points.

Stroke volumes quantified by baseline ASPECTS on admission non-contrast CT, as well as the neurological functional status, measured by the modified Rankin Scale (mRS) at discharge and at the 3-month follow-up, showed significant differences between the groups (Table 1; Figure 1). The mortality rate was significantly higher in the HT group, both at discharge: 33% vs. 18%, *p* = 0.038, and at follow-up: 42% vs. 12%, *p* < 0.001.

**Table 1:** Comparative radiological and functional outcome data of the analyzed patients.

Parameters	Overall, *N* = 150^a^	Control group, *N* = 95	HT group, *N* = 55	*p*-value^b^
ASPECTS–admission CT				**0.004**
Mean (SD)	8 (2)	9 (2)	7 (3)	
Median (IQR)	9 (3)	10 (2)	8 (4)	
Range	0–10	0–10	0–10	
ASPECTS–follow-up CT				**<0.001**
Mean (SD)	6 (3)	7 (2)	4 (3)	
Median (IQR)	6 (4)	8 (3)	4 (4)	
Range	0–10	0–10	0–10	
Discharge mRS score				**<0.001**
Mean (SD)	4 (1)	4 (1)	5 (1)	
Median (IQR)	4 (2)	4 (1)	4 (2)	
Range	1–6	1–6	2–6	
3 months follow-up mRS score				**<0.001**
Mean (SD)	4 (2)	3 (2)	4 (2)	
Median (IQR)	3 (2)	3 (2)	4 (3)	
Range	0–6	0–6	2–6	

a *n* (%); Data are presented as mean (standard deviation), median (interquartile range), and range.

b Pearson’s Chi-squared test; Fisher’s exact test; Two Sample *t*-test. Bold values indicate statistically significant differences (*p* < 0.05) between groups.

We have registered the rate of revascularization therapy among the analyzed patients, by intravenous thrombolysis (IVT), endovascular thrombectomy (EVT) or both. To note, 21 out of 150 patients (14%) received such treatment: 13 patients (24%) in the research group and 8 patients (8.4%) in the control group, with the difference reaching statistical significance (*p* = 0.01). The patients from the control group received exclusively intravenous thrombolysis, while in the HT-group 4 patients had IVT, 5 EVT and 4 received IVT followed by EVT. We could compare the onset-to-reperfusion time only for the IVT patients (8 patients in each group), given that EVT was performed solely in the HT-group. No statistically significant difference was found between the two cohorts: 152.8 min (SD = 26.2; range: 125–193 min) in HT-group vs. 167.1 min (SD = 46.5; range: 120–260 min), in the control group, *p* = 0.48.

Univariate logistic regression analysis revealed that AF, low ASPECTS and application of revascularization therapy were significantly associated with higher risk of HT (Table 2). AF and low ASPECTS were negatively associated with functional recovery at discharge and 3 months follow-up, while revascularization therapy was associated with better discharge status, and did not impact the 3 months follow-up status, as detailed in Table 2.

**Table 2:** Correlation between the analyzed parameters, the risk for hemorrhagic transformation, functional recovery at discharge and 3 months post-stroke.

Parameters/type of analysis	Univariate analysis			Multivariate analysis		
	OR	95% CI	*p*-value	OR	95% CI	*p*-value
Correlation with the HT						
Atrial fibrillation	3.51	1.63–7.94	0.002	15.86	3.44–95.15	0.001
Admission ASPECTS	0.77	0.64–0.91	0.003	0.91	0.71–1.16	0.422
IVT/EVT	2.76	1.05–7.62	0.042	5.18	1.19–25.38	0.033
Correlation with discharge mRS						
Atrial fibrillation	4.91	1.38–23.02	0.022	17.19	0.56–1.44	0.142
Admission ASPECTS	0.52	0.22–0.87	0.052	0.65	0.21–1.49	0.364
IVT/EVT	0.515	0.14–2.49	0.355	0.02	0.001–0.75	0.072
Correlation with follow-up mRS (3 months)						
Atrial fibrillation	3.68	1.41–10.21	0.009	4.17	0.58–37.76	0.172
Admission ASPECTS	0.65	0.42–0.91	0.030	0.615	0.33–0.98	0.073
IVT/EVT	1.06	0.33–4.06	0.931	0.18	0.03–0.91	0.050

OR, Odds Ratio; CI, Confidence Interval; IVT, Intravenous Thrombolysis; EVT, Endovascular Thrombectomy; mRS, modified Rankin Scale.

A following multivariable logistic regression analysis showed that AF, admission ASPECTS and revascularization therapy remained a significant predictor for HT (Table 2). For functional recovery, low ASPECTS remained a bad predictor, while revascularization therapy correlated with better outcome both at discharge and follow-up (Table 2).

Based on the multiple regression model, we have calculated the log (odds) of having HT for each patient. The coefficients for the model were statistically significant for admission ASPECTS (*p* = 0.00071), AF (*p* = 0.00394), and revascularization therapy (*p* = 0.00739). The model’s residual deviance was 143.85, and the Akaike Information Criterion (AIC) was 151.85, indicating a good model fit.

Other common cardiovascular risk factors (high blood pressure and diabetes mellitus) did not show significant rate differences between groups (HBP proportion in HT-group vs. non-HT group: 98% vs. 97%, *p* > 0.99; DM rate in HT-group vs. non-HT group: 33% vs. 36%, *p* = 0.7), neither correlated with HT in univariate and multivariate models, being excluded from the final predictive analysis.

**Figure 2:**
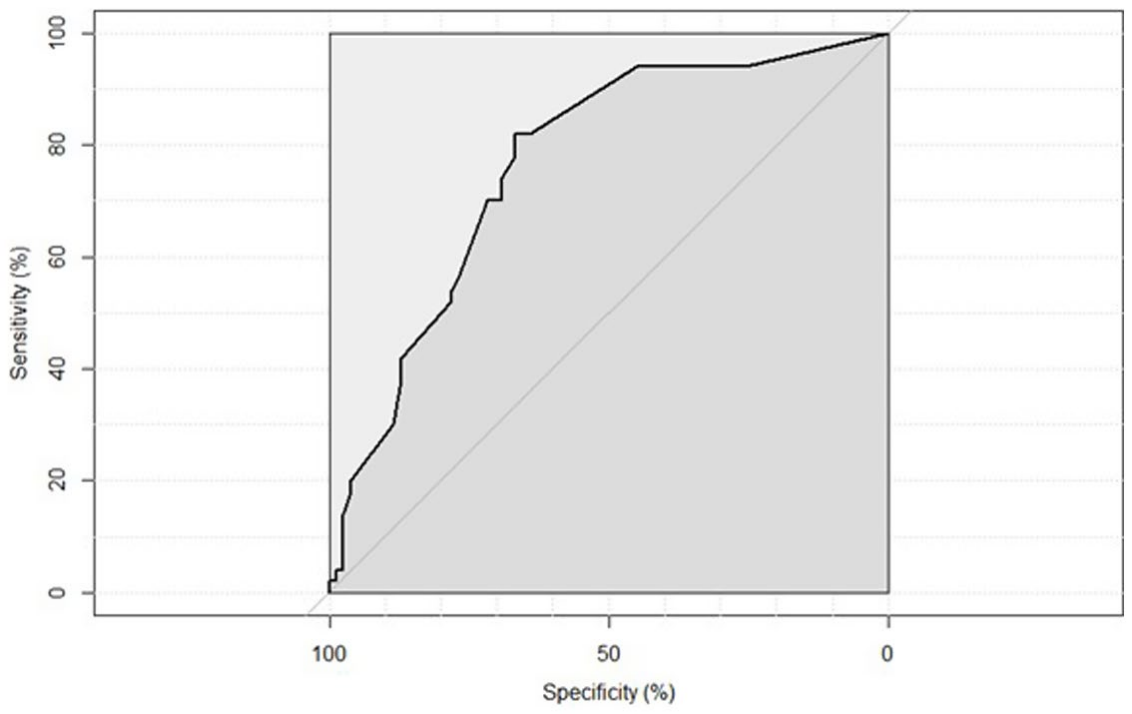
ROC curve illustrating the predictive value of the combined model including atrial fibrillation, revascularization therapy, and ASPECTS score for the occurrence of hemorrhagic transformation. AUC = 76.51% (95% CI: 68.16–84.86%).

Receiver Operating Characteristic (ROC) curve analysis demonstrated that the combined presence of atrial fibrillation (AF), administration of revascularization therapy, and lower admission ASPECTS scores showed a reasonable predictive value for HT complication development, with the area under the curve (AUC) of 76.51% (95% CI: 68.16–84.86%) (Figure 2).

## Discussion

The exact mechanisms of hemorrhagic transformation development in ischemic stroke patients are not well established, hence the lack of predictive and preventive tools of this frequent complication of ischemic stroke (10). Literature data stresses out numerous possible risk factors for HT (11), clinical, radiological, and serological (12, 13).

Our study included 150 acute ischemic stroke patients with 55 developing HT post-stroke. We identified several clinical and imaging parameters associated with an increased risk of hemorrhagic transformation (HT) during hospitalization. Our findings reinforce the multifactorial nature of HT and highlight the potential utility of early predictors such as atrial fibrillation (AF), low ASPECTS scores, and the use of cerebral revascularization therapies.

Cardioembolic strokes are strongly associated with HT according to previous studies (14, 15). Our results confirm these findings, with a higher rate of cardioembolic type of ischemic stroke in patients with HT–52.73%, compared to the control group–35.79%.

Moreover, our data confirm AF as a major independent predictor of HT and poorer functional recovery. Atrial fibrillation (AF) presented statistically significant differences between groups, being more common in patients with ulterior HT development (73% versus 47%, *p* = 0.003), similar to the literature data (16), and presence of AF was associated with a 3.5-fold increase of the odds for developing post-stroke HT in patients with AF (OR = 3.5, *p* = 0.004). Consecutively, atrial fibrillation correlated with unfavorable outcomes at discharge by higher mRS score (OR = 4.9, 95% CI: 1.38–23.02, *p* = 0.022), as well as at 3 months follow-up (OR = 3.68, 95% CI: 1.41–10.21, *p* = 0.009), corresponding to other published data, which indicate that AF not only increases the risk of stroke by about 5 times but is also doubles the mortality rate in these patients (17). Although 85 patients in our study had a known diagnosis of atrial fibrillation (40 patients in the hemorrhagic transformation group and 45 patients in the control group), only 20 (10 per group) were receiving warfarin prior to stroke. Besides, only one patient in each group had optimal anticoagulation, making difficult to assess possible correlation between previous anticoagulation and post-stroke risk oh HT.

High blood pressure is constantly reported to worsen post-stroke evolution; appropriate anti-hypertensive treatment being associated to reduced complications’ rate and better outcome (18). Our data analysis did not reveal such correlations, findings aligning with other research on paradoxical evolution in patients with well controlled HBP (19). Similarly, diabetes mellitus can aggravate the clinical state of patients with cerebral infarction, according to literature data (20), statement that we could not confirm in our study. These results can be explained but similar rates of HBP and DM in the analyzed groups.

According to literature data, the ASPECTS score correlates inversely with the risk of HT (21). In our research study, patients had significantly lower ASPECTS scores (calculated on the admission CT scans) in the HT cohort compared to the control group, *p* = 0.004, consistent with findings in similar research (22). Additionally, regression analysis emphasized the relationship between higher ASPECTS scores at admission and the reduction in HT risk (OR = 0.7, *p* = 0.001), as well as the correlation with favorable outcomes at discharge (OR = 0.52, *p* = 0.05), and at 90 days post-stroke (OR = 0.65, *p* = 0.03). Therefore, a low ASPECTS was negatively associated with the risk for HT, the functional recovery at both discharge and 3 months, underlining its dual prognostic utility.

In our analysis of haemostasis laboratory parameters, only INR showed a statistically significant difference between groups, with lower values observed in patients who developed HT compared to controls (mean INR 1.27 vs. 1.38, *p* = 0.003). This finding appears paradoxical, as elevated INR is typically associated with an increased risk of hemorrhagic events (23). However, both univariate (OR = 0.1, 95% CI: 0.02–0.5, *p* = 0.01) and multivariate regression analyses (OR = 0.9, 95% CI: 0.01–0.75, *p* = 0.033) in our study revealed an inverse relationship between admission INR and the risk of HT. A potential explanation may be that a low INR correlates with a prothrombotic state, which can facilitate the formation of larger thrombi, potentially leading to more severe cerebral infarcts (24). Notably, patients who developed HT in our study, had significantly lower ASPECTS scores at baseline compared to those without HT (*p* = 0.004), supporting the above-presented hypothesis.

Revascularization therapies were more frequent in the HT group (24% in the HT group and 8.4% in the control group, *p* = 0.01), confirming previous studies results (25). To note, revascularization therapies, while more frequent in the HT group, were associated with improved short-term outcomes and were not predictive of worse long-term recovery. The obtained results correspond to current literature data about the complex interplay between therapeutic benefit and hemorrhagic risk (26).

Although the overall number of patients receiving reperfusion therapy in our cohort was modest (21 out of 150 patients–14%: 13 patients (24%) in the research group and 8 patients (8.4%) in the control group, *p* = 0.01), the incidence of HT was significantly higher among those treated with intravenous thrombolysis and/or endovascular therapy. To note, the patients from the control group received exclusively intravenous thrombolysis, while in the HT-group 4 patients had IVT, 5 EVT and 4 received IVT followed by EVT. Concordant to prior evidence, our data suggests, indirectly, that large vessel occlusion is a key contributor to HT risk, especially when reperfusion is achieved (27). The underlying mechanism likely involves blood–brain barrier disruption due to ischemia–reperfusion injury which is amplified by thrombolytics (28).

The multivariable logistic model combining AF, ASPECTS score, and revascularization therapy demonstrated good predictive performance for HT (AUC = 76.51%), supporting the utility of integrated risk profiling. While not definitive, these findings suggest that individualized risk stratification using routinely available clinical and imaging variables may help guide therapeutic decisions and post-treatment monitoring. Nonetheless, the study adds valuable prospective evidence on the real-world predictors of HT and functional outcomes in patients treated across a broad therapeutic spectrum.

Limitations on the study comprise several key aspects, including the single-center design, the moderate sample size, and the lack of advanced imaging modalities (e.g., MRI) that might further refine risk assessment. Susceptibility-weighted imaging MRI was not routinely available in our research, therefore evaluation for cerebral microbleeds or cerebral amyloid angiopathy, which may have contributed to the risk of HT, could not be realized. Further multicentric research on larger cohorts is mandatory to confirm and validate the obtained results.

## Conclusion

Our study emphasizes that ASPECTS score, atrial fibrillation, and revascularization therapy are important predictors of hemorrhagic transformation in acute ischemic stroke. These results support the use of a combined clinical-imaging approach for risk stratification of HT in AIS patients and highlight the need of continued improvement of predictive strategies.

## Data Availability

The raw data supporting the conclusions of this article will be made available by the authors, without undue reservation.
